# Melatonin Rinsing Treatment Associated with Storage in a Controlled Atmosphere Improves the Antioxidant Capacity and Overall Quality of Lemons

**DOI:** 10.3390/foods13203298

**Published:** 2024-10-17

**Authors:** Mengjiao Yang, Enlan Zheng, Ziqin Lin, Ze Miao, Yuhang Li, Shiting Hu, Yanan Gao, Yuqian Jiang, Lingling Pang, Xihong Li

**Affiliations:** 1 State Key Laboratory of Food Nutrition and Safety, College of Food Science and Engineering, Tianjin University of Science and Technology, Tianjin 300457, China; yangmj202202@163.com (M.Y.); zhengenlan@163.com (E.Z.); linziqin1998@163.com (Z.L.); miaoze1223@126.com (Z.M.); 18233236664@163.com (Y.L.); a15032404727@163.com (S.H.); 18722497117@163.com (Y.G.); jiangyuqian@tust.edu.cn (Y.J.); 2 College of Biosystems Engineering and Food Science, Zhejiang University, Hangzhou 310058, China; panglingling2015@163.com

**Keywords:** lemon, combined treatment, antioxidant, storage quality

## Abstract

Antioxidant capacity is one of the most important biological activities in fruits and vegetables and is closely related to human health. In this study, ‘Eureka’ lemons were used as experimental materials and stored at 7–8 °C MT (melatonin, 200 μmol, soaked for 15 min) and CA (controlled atmosphere, 2–3% O_2_ + 15–16% CO_2_) individually or in combination for 30 d. The changes in lemon fruits’ basic physicochemical properties, enzyme activities, and antioxidant capacities were studied. Comparing the combined treatment to the control, the outcomes demonstrated a significant reduction in weight loss, firmness, stomatal opening, and inhibition of polyphenol oxidase (PPO) and peroxidase (POD) activities. Additionally, the combined treatment maintained high levels of titratable acidity (TA), vitamin C (VC), total phenolic content (TPC), and antioxidant capacity and preserved the lemon aroma. Meanwhile, the correlation between fruit color, aroma compounds, and antioxidant capacity was revealed, providing valuable insights into the postharvest preservation of lemons. In conclusion, the combined treatment (MT + CA) was effective in maintaining the quality and antioxidant capacity of lemons.

## 1. Introduction

Lemons are high in dietary fiber, vitamin C, and minerals, which possess strong antioxidant properties and confer benefits to human health [1]. However, lemons are highly susceptible to discoloration, softening, dry wrinkling, and rotting during storage due to mechanical damage, improper storage conditions, and other factors, resulting in tissue aging and a decline in organoleptic quality and causing significant economic losses [2,3]. The main methods reported to improve the storage quality of lemons such as melatonin, beeswax, controlled atmosphere, chitosan, 1-MCP, and methyl jasmone treatment [4,5,6,7,8,9].

Controlled atmosphere (CA) treatment has been widely used to preserve a variety of fruits and vegetables, such as lentils, blueberries, avocados, plums, and apples [10,11,12,13,14]. It is thought to be an effective way to postpone postharvest fruit ripening and senescence. The postharvest respiration of lemons was inhibited by CA, and transpiration was also hindered by it, which delayed the degradation of lemon chlorophyll and delayed the aging process of lemons [15]. CA used in the preservation of fruits has the unique advantages of being non-residual, effective, energy-saving, and environmentally friendly. However, long-term CA storage promotes anaerobic respiration, leading to elevated levels of acetaldehyde and ethanol in fruits. This process not only results in undesirable odors but also increases the production of reactive oxygen species (ROS) [16,17]. Therefore, additional treatments in combination with CA are required to reduce unwanted odor. By chance, melatonin (N-acetyl-5-methoxytryptamine, MT), an indoleamine, serves as a multi-regulatory compound in living organisms. It significantly contributes to the regulation of ROS, reactive nitrogen species (RNS), and other free radicals, alongside harmful oxidative molecules found in plant cells. [18,19,20]. MT, as a naturally occurring antioxidant, has the potential to meet the developmental requirements for the safe and healthy preservation of fruit, particularly in the context of green and organic practices. Its versatility lends itself to a multitude of prospective applications. Recently, the role of MT in the postharvest preservation of fruits and vegetables has gradually attracted attention, and it has been described that MT application can maintain higher antioxidant activity, delay fruit senescence in sweet cherries, blackberries, and jujubes [21,22,23], and reduce the degradation of chlorophyll in fruits and vegetables, such as bell peppers, passion fruits, and cucumbers, to maintain their good color [24,25,26]. In addition, MT-treated lemons had an increased total phenolic content and better organoleptic qualities [4]. Meanwhile, the postharvest disease incidence and decay rate of blueberries, grapes, tomatoes, and other fruits can be effectively reduced by MT [27,28,29,30], which prolongs the storage time of fruits and vegetables.

To the best of our knowledge, many previous studies have concentrated on the individual impacts of MT or CA on lemon preservation quality. However, there is a lack of studies that investigate the combined effects of MT and CA. In this study, a combination of MT and CA treatments was exogenously applied to postharvest lemons to analyze the effects of MT and CA treatments on lemon physiological characteristics, oxidative defense enzyme activities, and antioxidants and to enhance the shelf life and quality of lemons.

## 2. Materials and Methods

### 2.1. Materials and Treatments

The ‘Eureka’ lemons analyzed in this research were cultivated and picked at peak commercial maturity in October 2023, in the Wanzhou District of Chongqing, China (30°24′25” N, 107°55′22” E). These lemons were placed in commercial-grade plastic stacking containers and shipped to Tianjin University of Science and Technology within 24 h using cold chain logistics, insulated with cold packs, and covered with blankets. Following a 24 h pre-cooling period at 7–8 °C and relative humidity (RH) of 90–95% in a refrigerated environment, lemons of uniform size (80–100 g per fruit), ripeness, and free of defects and diseases were selected. The lemons were then divided into four groups. Each group underwent a thorough rinse with water and subsequently dried using sterile gauze. Samples stored at 7–8 °C and 90–95% RH for 30 d were set up as the Control group. Treatment group 1 was placed directly in the gas conditioning bottle (2–3% O_2_, 15–16% CO_2_), recorded as CA; Treatment group 2 was immersed in a 200 μmol melatonin solution for 15 min, recorded as MT; and Treatment group 3 was immersed in a 200 μmol melatonin solution for 15 min before being placed in a gas conditioning bottle, recorded as MT + CA. All treated fruits were stored at 7–8 °C and 90–95% RH for 30 d. Physiological changes were measured and recorded in random samples taken at 5-day intervals (0, 5, 10, 15, 20, 25, and 30 d).

### 2.2. Color Measurement and Browning Index

The lemon of each treatment group was measured using a colorimeter (HP-200, Hanpu Photoelectric Technology Co., Ltd., Shanghai, China), and three different positions were selected at the lemon equator. The L*, a*, and b* values of each point were recorded. The chroma C* is calculated with the Formula (1) [31]. The chlorophyll content was determined by weighing 1 g of the sample into a mortar, adding 2–3 mL of 80% acetone to study into a homogenate until the tissue turned white, leaving it to extract for 3–6 min, and determining the absorbance at 652 nm.

(1) $$C^{*}\;=\;\sqrt{{a^{2}}^{*}+{b^{2}}^{*}}$$

### 2.3. Fruit Quality

Weight loss was calculated by subtracting the final weight from the initial weight on the storage day, and the results were presented as % (weight in weight). Lemon hardness was determined using a portable digital fruit hardness tester (GY-4, Zhejiang Top Instrument Co., Ltd., Hangzhou, China) with a 3.5 mm cylindrical probe and expressed as N [13]. The soluble solids content (SSC) was measured using a digital refractometer (PAL-1, Atago Co., Tokyo, Japan), and the results were presented as %. The titratable acidity (TA) content of lemon was assessed by dropping 10 mL of lemon juice to reach PH 8.2 using the 0.1 mol L^−1^ NaOH solution, and the results were expressed as citric acid in %. The vitamin C (VC) was determined by titrating the 2,6-dichlorophenol-indophenol solution until a slight red color appeared and did not fade away for 15 s after extracting the extracts with a 20 g L^−1^ oxalic acid solution [32].

### 2.4. Enzyme Activity

The enzymatic activities of polyphenol oxidase (PPO) and peroxidase (POD) were assessed utilizing the methodology outlined by Jiang et al. [33]. Briefly, 3 g of lemon was weighed and added to 3 mL of 100 mM, PH 5.5 phosphate-buffered solution (PBS, 4% polyvinylpyrrolidone, and 1% Triton X-100). This mixture underwent vigorous mixing in an ice water bath. Following that, centrifugation occurred at 10,000× *g* for 30 min at 4 °C to collect the supernatant as the enzyme extract for subsequent analysis. To evaluate PPO activity, 60 μL of the enzyme extract was reacted with 240 μL of a 50 mM acetic acid-sodium acetate buffer (PH 5.5) and 60 μL of 50 mM catechol, with the absorbance measured at 420 nm. For POD activity assessment, 30 µL of the enzyme extract was combined with 180 µL of 25 mM guaiacol and 30 µL of 500 mM H_2_O_2_, followed by absorbance evaluation at 470 nm.

The Phenylalanine Ammonia-Lyase (PAL) assay was conducted by extracting the sample with 40 g L^−1^ PVP, 2 mmol L^−1^ EDTA, and 5 mmol L^−1^ *β*-mercaptoethanol at a low temperature, centrifuging at 12,000× *g* for 30 min, and then combining 50 mmol L^−1^ boric acid buffer and 20 mmol L^−1^ L-phenylalanine, and the mixture was held for 10 min to determine the initial value at 290 nm. Subsequently, the mixture was held for 60 min to determine the termination value. The initial value was determined at 290 nm after the 10 min holding period, and the termination value was determined after an additional 60 min of holding [34].

Malondialdehyde (MDA) was determined based on prior work [35]. Then, 1 g of lemon was ground with 5 mL of trichloroacetic acid (TCA, 100 g L^−1^) and centrifuged for 20 min at 10,000× *g*. Next, 2 mL of the supernatant was combined with 2 mL of thiobarbituric acid (TBA, 6.7 g L^−1^), and then placed in a boiling water bath for 20 min. After cooling, the mixture was centrifuged once more, and the absorbance of the supernatant was read at 450 nm, 532 nm, and 600 nm. The MDA was measured in µmol kg^−1^.

### 2.5. Antioxidant Capacity

The total phenolic content (TPC) was assessed using the Folin–Ciocalteu method, with minor modifications as previously described [36]. Then, 5 g of the freeze-dried lemon sample was mixed with 25 mL of methanol, sonicated for 30 min, and centrifuged at 10,000× *g* for 10 min, and the supernatant was used for TPC determination. In brief, a 96-well plate was used, containing 25 μL of supernatant and 125 μL of Folin reagent (Shanghai Yuanye Biotechnology Co., Ltd., Shanghai, China) per well. The plate was allowed to incubate for 10 min at room temperature. Subsequently, 125 μL of a 10% Na_2_CO_3_ solution was added, and the reaction proceeded for 30 min. A spectrometer (Spectra Max 190, Molecular Devices Corporation, California, USA) was used to measure the absorbance at 765 nm using methanol as a blank. Based on gallic acid, a standard curve was constructed, and the results were expressed in g kg^−1^ units of a gallic acid equivalent.

The DPPH radical scavenging activity was assessed using a previously reported method [37]. Briefly, 25 μL phenolic extract and 200 μL DPPH solution (350 μM methanol) were added to a 96-well plate, allowing it to remain in darkness for 4 h at room temperature. Absorbance was read at 517 nm. The DPPH scavenging activity was determined from a standard curve of Trolox equivalents and reported in mmol kg^−1^.

A hydrogen peroxide detection kit (Beijing Solarbio Science & Technology Co., Ltd., Beijing, China) was used to detect the H_2_O_2_ content, and the results were expressed as µmol kg^−1^.

The catalase (CAT) activity was determined as described by Li et al. [35]. Briefly, 5 g of powder was extracted with 5 mL of PBS (100 mM, pH 7.5) in an ice bath. After centrifugation at 4 °C for 30 min at 10,000× *g*, the supernatant (0.5 mL) was mixed with 2.9 mL of H_2_O_2_ (20 mmol), and the absorbance was measured at 240 nm every 30 s for 6 min.

### 2.6. Scanning Electron Microscopy (SEM)

The ultrastructure of lemon was observed by the scanning electron microscopy method previously described [7], with slight modifications. Lemons (approximately 10.0 × 10.0 × 1.0 mm^3^) were immersed in a 2.5% formaldehyde solution at 4 °C for 12 h. They then received three rinses with 0.2 M PBS before fixation in a 2% osmium tetroxide solution for 1 h. Following this, the samples underwent another three rinses with PBS. Subsequently, dehydration occurred through a graded ethanol series (30%, 50%, 70%, 80%, 90%, and 95% *v*/*v*), after which they were dried and placed in a vacuum desiccator overnight. Finally, the sample was gold-plated for 2 min with a thickness of 2 nm using a sputtering coating machine. Then, the scanning electron microscope (SU8010, Hitachi Company, Tokyo, Japan) was used for observation.

### 2.7. Aroma Analysis by E-Nose

Lemon volatiles were analyzed for different storage times utilizing a PEN E-nose instrument (Winmuster Airsense Analytics Inc., Schwerin, Germany) [38]. Ten metal oxide semiconductor sensors detected various compounds and produced corresponding response values. An amount of 3 g of grated lemon was combined with 3 mL of saturated sodium chloride solution in a sealed 20 mL glass vial and warmed for 20 min in a 40 °C water bath; then, we conducted electronic nose detection. Headspace gas was continuously injected into the detector for 100 s until the signal line stabilized. Response values were recorded over time and were processed using WinMuster electronic nose software (WinMuster PEN v 1.6.2.18). Three replicates of each sample were analyzed using the electronic nose.

### 2.8. Statistical Analysis

The experiment was randomized, and each treatment was replicated three times. Statistical analysis employed IBM SPSS Statistics 22.0 software (IBM Corp., Armonk, NY, USA). The mean values of the various treatments underwent comparison through a multiple-range test (Tukey test), establishing the significance level threshold at *p* < 0.05.

## 3. Results and Discussion

### 3.1. Effect on Color and Chlorophyll Content of Lemon Fruit

**Figure 1:**
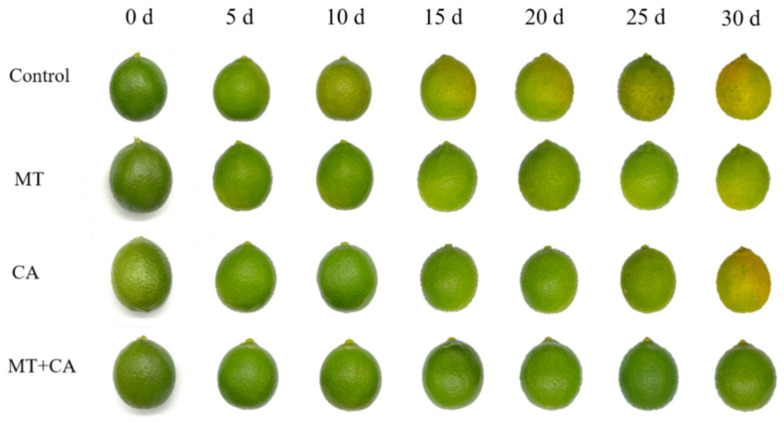
The visual appearance of lemons treated with MT and/or CA.

The peel color is typically linked to the intrinsic qualities of the fruit, such as flavor and texture, which can impact consumer purchasing decisions [39]. Green lemons undergo bio-enzymatic oxidation during storage, resulting in a gradual increase in the degree of yellow coloration and a concomitant yellowing of the peel, which may even progress to browning in some areas [40]. Research indicates that the shift in lemon fruit color from green to yellow results from changes in the levels of chlorophyll and carotenoids. Several studies have demonstrated that melatonin markedly attenuates chlorophyll degradation in broccoli, tomatoes, and cabbage, while also exerting a beneficial influence on chlorophyll-like and carotenoid levels and photosynthesis [41,42,43,44]. In addition, the postharvest MEL treatment of ‘Fino’ lemon fruits was recently reported to have a positive effect on the reduction in fruit color change, weight loss, and total acidity loss [4]. Our results demonstrated that melatonin had a comparable impact, with the postharvest association of MT and CA, as illustrated in Figure 1, markedly decelerating the fruit degreening process. The use of melatonin diminished the growth rate of the C* value and chlorophyll levels in lemon peels (Table 1). The combined treatment maintained the green color for a longer period than the Control group.

### 3.2. Effect on Quality Characteristics of Lemon

**Figure 2:**
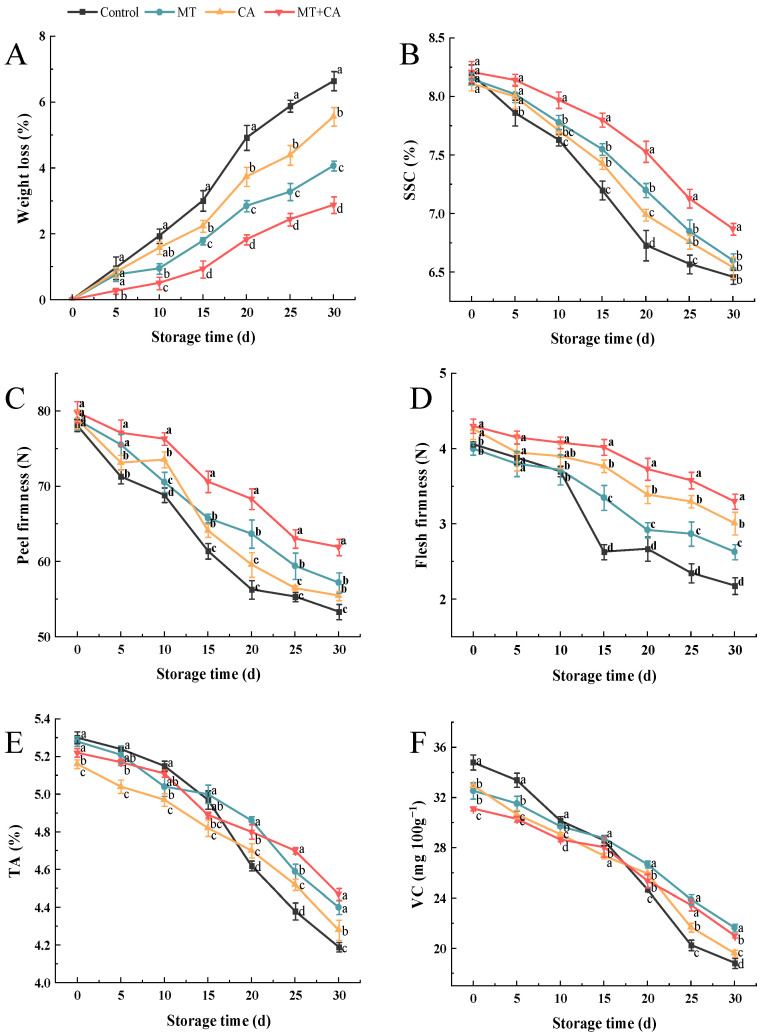
Basic physicochemical properties of MT or/and CA-treated lemons during 30 d of cold storage at 7–8 °C versus control. (**A**) Weight loss; (**B**) SSC; (**C**) peel hardness; (**D**) flesh hardness; (**E**) TA; (**F**) VC. Values represent means ± SD in triplicate, and different letters denote significant differences compared to the control during the same storage time at the *p* < 0.05 level.

Weight loss in fruits and vegetables during storage primarily results from water loss through transpiration. This process significantly impacts the quality of lemons, leading to notable degradation [45]. Following harvest, lemons continue to lose water in vapor form through stomata and the surface layer, contributing to reductions in fruit weight [46]. Physiological water loss plays a critical role in determining fruit quality and susceptibility to various postharvest diseases. The effects of various treatments on lemon weight loss can be observed, showing a consistent increase across all groups (Figure 2A). On day 30, the Control group experienced the highest weight loss, reaching 6.64%, while the MT + CA treatment group had the least at 2.88%. Our results and previous work suggest that MT application can delay water loss during cold storage [47]. Wang et al. [21] and Shah et al. [22] demonstrated that the postharvest application of melatonin reduced weight loss in sweet cherries and blackberries. The reduced weight loss in lemons treated with MT may stem from decreased levels of MDA content and H_2_O_2_ free radicals. In contrast, the diminished weight loss in CA-stored lemons likely results from lower ripening and respiration rates [48]. Given that all lemons underwent storage under identical temperature and relative humidity conditions, the minimal weight loss observed in the MT + CA treatment can be linked to the reduced respiration rate and limited stomatal opening, as evidenced by SEM observations.

Softening significantly contributes to quality degradation and reduced shelf life in both menopausal and non-menopausal fruits. It should be noted that all lemons were at the same stage of ripening and the peel firmness diminished over time as storage duration increased, as illustrated in Figure 2C. The peel hardness of the Control group was 78.08 ± 0.78 N at harvest and decreased to 53.36 ± 1.01 N on day 30. After storage, the peel hardness in the MT, CA, and MT + CA groups surpassed that of the Control group by 7.23%, 3.97%, and 16.06%, respectively. The trend in decreasing pulp and peel hardness persisted (Figure 2C), likely due to delayed cell wall degradation [49]. Research indicates that MT treatment positively influences the preservation of fruit firmness and quality, particularly in mangoes, bananas, and pomegranates [50,51,52]. Furthermore, low temperatures, minimal O_2_ levels, and elevated CO_2_ concentrations effectively inhibited water migration, thereby maintaining the quality of stored fruits. In our work, MT + CA treatment significantly slowed down the decrease in hardness, especially in lemon peel, and effectively maintained the hardness of the fruit.

The TA, SSC, and VC concentration are key physiological indices for assessing the intrinsic quality of fruits. The SSC can be used to quantify fruit ripening and senescence. As illustrated in Figure 2B, the SSC of all groups exhibited a declining trend. The reduction in SSC may be attributed to the depletion of nutrients through catabolic processes during storage or due to water loss [3,53], indicating that lemons undergo a gradual process of senescence. The SSC of control, MT, CA, and MT + CA exhibited a decline of 1.73%, 1.55%, 1.57%, and 1.34%, respectively, over the course of 30 d of storage. Previous studies have demonstrated that MT maintains higher SSC in blackberry, nectarine, and blueberry [22,54,55]. Similarly, Tian et al. [56] found that SSC was reduced with the increase in storage time and that CA was superior to MAP and RA in preserving fruit SSC. Therefore, the elevated SSC of the combined treatments may be attributed to the delayed onset of senescence in lemons resulting from the application of MT and CA.

Citric acid is the primary organic acid found in lemons. The results are displayed in Figure 2E, where TA gradually decreased, which is consistent with the findings of Serna-Escolano et al. [57]. The difference in the TA change between the MT and CA groups was not significant, but it was significantly different from the other two groups, with only a 0.75% decrease in MT + CA. It has been noted that MT can mitigate the acidity drop in other fruits, including pears, sweet cherries, and navel oranges [49,58,59]. Fruits kept in CA had a high TA level, which was linked to a slower ripening process and a decreased respiration rate [60]. The concentrations of O_2_ and CO_2_ were critical in causing the samples to ripen more slowly [61]. The reduced decarboxylation of organic acids, including citric and malic acids, which are frequently utilized as substrates for respiratory enzyme processes, in fruit exposed to high CO_2_ and low oxygen during storage could also be the cause of this outcome [62].

A higher VC content in lemons indicates a higher nutritional value and showed a decreasing trend during storage. As shown in Figure 2F, after the experiment, the VC content of the control fruits dropped dramatically from 34.8 mg 100 g^−1^ to 18.84 mg 100 g^−1^, while the VC content of the MT + CA-treated fruits only declined to 21.02 mg 100 g^−1^. After 15 days of storage, it was discovered that the VC content of the treatments drastically dropped. Similarly, Selcuk et al. [63] and Tian et al. [64] reported a trend toward a decline in VC levels in wolfberry and sweet cherry fruits throughout storage. The use of various organic acids during fruit respiration or their potential conversion to sugars may be the cause of the drop in VC under storage conditions [65]. VC, as a major antioxidant, directly scavenges ROS, and the higher VC level indicates the superior antioxidant capacity of MT + CA-treated lemon.

### 3.3. Synergistic Promotion of TPC and Inhibition of ROS

**Figure 3:**
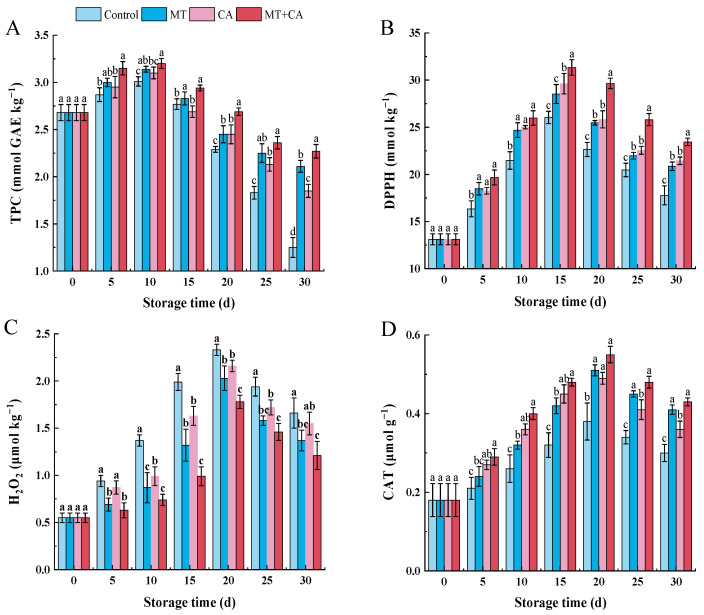
Effects of MT, CA, or combined treatments on ROS production and membrane peroxidation compared with the control within the same storage time. (**A**) TPC, (**B**) DPPH scavenging capacity, (**C**) H_2_O_2_, (**D**) CAT activity. Values were expressed in the mean ± SD (n = 3), and different letters suggest significant differences within different treatments compared with control at *p*  <  0.05.

The phenolic content of lemons is one of the main reasons for their antioxidant and anti-tumor properties. The evolution of TPC in lemons for 30 d is shown in Figure 3A, with a general trend of increasing and then decreasing in all groups. Among them, the MT treatment reached the highest value of 3.2 mmol GAE kg^−1^ on day 10, consistent with studies by Ma, Q et al. [58] MT also showed an accumulation of TPC during the storage of oranges, thus maintaining the postharvest quality. After 10 d of storage, the TPC started to decrease in all treatments, and the Control group saw the fastest decline; on day 30, there was a noticeable difference. Because of the loss of astringent flavor and changes in enzyme activity, phenolic degradation is responsible for the decrease in TPC [63,66]. Samples kept in CA may have high TPC levels because oxidation is inhibited and membrane leakage is decreased. POD and PPO may eventually bind to the contents due to compromised membrane integrity [65]. The role of MT and CA in the accumulation of TPC has been demonstrated in lemon and lychee, among others [9,58,65]. Lemons treated with MT + CA in the current study had greater TPC during cold storage, which could be related to increased activity of antioxidant enzymes such as CAT, weight loss, and decreased activity of PPO and POD enzymes.

The antioxidant activity of lemon fruits has received wide attention from consumers, and DPPH and H_2_O_2_ are commonly used indicators for evaluating antioxidant activity in vitro. As shown in Figure 3B,C, DPPH and H_2_O_2_ showed an increasing and then decreasing trend during lemon storage. The control fruits showed low DPPH scavenging capacity and high H_2_O_2_ accumulation, reaching a maximum value of 2.33 μmol g^−1^ Trolox after 20 d. In comparison, MT+CA significantly improved the scavenging capacity of DPPH and decreased H_2_O_2_ damage to the fruits. The oxidation and degradation of TPC may be the cause of the loss of antioxidant activity in fruit. TPC was a major contributor to the total non-enzyme antioxidant ability, which had a negative correlation with the senescence of fruit [67]. Thus, lemons treated with MT+CA showed an increased ability to scavenge DPPH, potentially linked to increased TPC levels. These results are consistent with several other reports that melatonin application significantly reduced H_2_O_2_ in stored peaches and cassava [68,69].

Fruits treated with MT, CA, and co-treatment had significantly higher CAT activity than control fruits during the cold storage period. CAT activity increased in all treatments up to 20 d of chilling, with the highest CAT activity in MT+CA treated fruits. On day 20, CAT activity decreased sharply (Figure 3D). MT mediates communication within and between plants and coordinates plant defense responses, including antioxidant systems. Cellular dezonalisation, accompanied by higher oxidase activity and senescence, improved CAT activity [70]. The slowing of lemon senescence and the retardation of oxidase activity after MT+CA treatment may be responsible for the increased CAT activity. This shows that CAT activity is a key factor in the reduction in oxidative stress by MT+CA treatment by reducing weight loss, H_2_O_2_ radicals, and MDA levels.

### 3.4. Enhancing the Activity of POD, PPO, and PAL and Inhibiting Membrane Peroxidation

**Figure 4:**
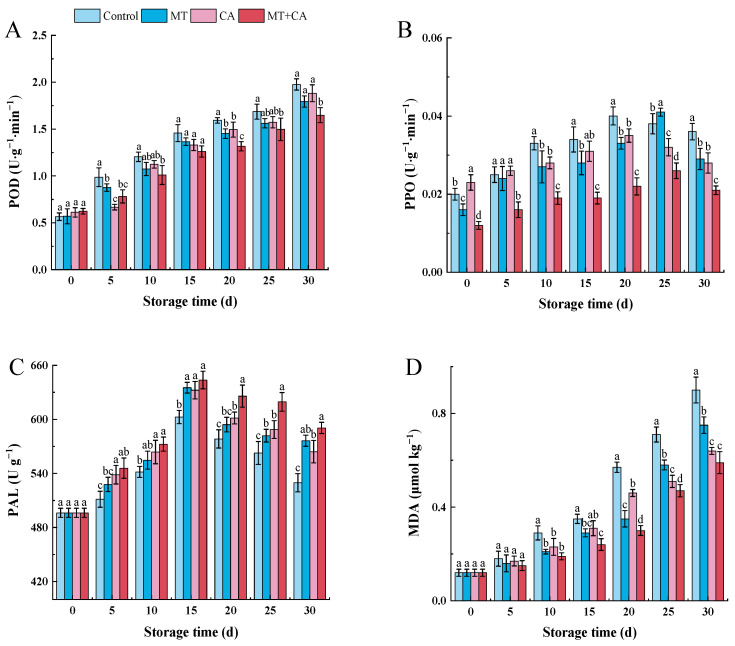
Changes in enzyme activities and membrane peroxidation in MT or/and CA-treated lemons during 30 d of cold storage at 7–8 °C versus control. (**A**) POD; (**B**) PPO; (**C**) PAL; (**D**) MDA. Values represent means ± SD in triplicate, and different letters indicate significant differences at the *p* < 0.05 level compared with the control during the same storage time.

Lemons accumulate more POD activity with storage time due to the cold storage environment. The control lemon had the highest activity after 30 d, at 1.976 U g^−1^ min^−1^. The POD activity of the lemon fruits treated with MT+CA, on the other hand, was significantly lower than that of the control fruits, at 1.648 U g^−1^ min^−1^ (Figure 4A). Comparatively, from day 25 to day 30, PPO activity exhibited an increasing trend followed by a decrease, as seen in Figure 4B. However, the low ethylene content and high citric acid content of all lemon fruits may be the reason for their minimal to negligible PPO activity. Lemons are common non-menopausal fruits that have a high citric acid content and comparatively little ethylene production [71,72]. The notable distinction between PPO and POD activities raises the possibility that distinct mechanisms are causing POD and PPO activities during cold storage [73]. The primary roles of POD and PPO enzymes are in phenolic catabolism and cellular catabolism brought on by weight loss and the buildup of ROS [74]. Thus, it is also possible to argue that the reduced POD and PPO activities in MT+CA-treated lemons might be the result of reduced ROS accumulation, weightlessness, and increased antioxidant capacity influenced by enzymatic and non-enzymatic antioxidants. These findings are in line with those of MT-treated blueberries that were previously discovered [55].

When stresses like low temperature are present, the PAL is essential to the metabolism of both the primary (mangiferic acid pathway) and secondary (phenylpropanoid pathway) types. There is substantial evidence that the phenylpropanoid pathway and PAL activity are connected to defense mechanisms in plants [75,76]. PAL activity increased significantly in the MT+CA treatment group, followed by the MT treatment group (Figure 4C). The results indicated that postharvest treatment MT+CA increased PAL activity, which was consistent with the increase in the cold tolerance of lemon fruits. It may be hypothesized that the enhancement of PAL activity is a defense mechanism against cold, which is related to PAL gene expression, and that MT might play a role in this process. Generally speaking, PAL, POD, and PPO are all involved in the expression of CI symptoms and are critical for phenolic metabolism [77].

MDA is frequently employed as a direct indicator of cellular oxidative damage and damage to membranes. The MDA content of lemon fruits increased during cold storage, and after the storage period, the MT+CA treatment dramatically reduced the MDA content of lemon fruits (Figure 4D). The MDA content of MT+CA-treated fruits on day 30 was 34.4% lower than that of control fruits. Mditshwa et al. [78] revealed that increased lipid peroxidation led to an increase in MDA content in all of the apples that were stored for a longer period. According to these results, membrane deterioration is caused by increased ROS-induced lipid peroxidation, which, in turn, causes an increase in MDA content, a lipid peroxidation product that indicates the true degree of ROS-induced membrane lipid peroxidation [67]. It is possible to link the decreased generation of MDA in MT+CA-stored fruits to a decrease in ROS accumulation and the inhibition of membrane peroxidation.

### 3.5. Regulation of Volatile Substances by MT and CA

**Figure 5:**
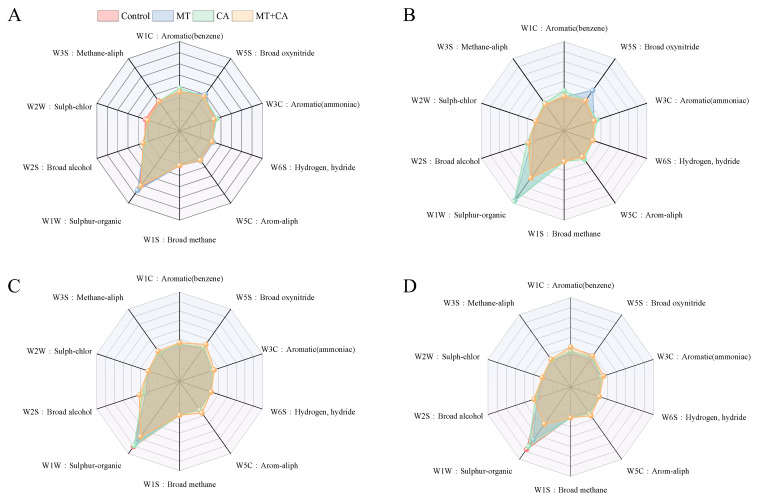
E-nose sensing on volatile organic compounds from MT, CA-treated, or mixed-treated lemons during storage at 7–8 °C compared to control. (**A**) E-nose sensing profiles on day 0, (**B**) day 5, (**C**) day 15, and (**D**) day 30. W1C: Aromatic, benzene; W5S: Broad range, oxynitride; W3C: Aromatic, ammoniac compounds; W6S: Hydrogen, hydride; W5C: Arom-aliph; W1S: Broad-methane; W1W: Sulfur-organic; W2S: Broad-alcohol; W2W: Sulph-chlor; W3S: Methane-aliph.

Fruit flavor and consumer preference are largely influenced by volatile compounds. The lemon flavor is the result of a combination of volatile (primarily olefins, ketones, alcohols, aldehydes, and esters) and non-volatile (primarily soluble sugars, amino acids, and organic acids) aroma compounds. In this study, the electronic nose volatile profiles of lemons were recorded and analyzed throughout storage as shown in Figure 5, and the perceptual responses of W3S, W6S, W1S, and W2W did not differ significantly between groups and did not vary much over storage time. The remaining sensors responded to each treatment to varying degrees, with sensor W1W being the most responsive, followed by W2S, W1C, and W5C. MT+CA showed higher responses than the control. According to the study of Zhong S. et al. [79], benzene and terpenoids were the major bound volatiles in Eureka lemon fruit. Aldehydes are thought to be among the most significant groups of volatile substances that affect the aroma of lemons [80]. Esters are a major source of flavor compounds in fruit and can give an indication of the flavor quality of the fruit; alcohols serve as solvents or carriers for the synthesis of aroma compounds [81]. It is noteworthy that the response of sensor W5S to the MT treatment decreased abruptly from day 5 to day 15.

### 3.6. Lemon Microstructure

**Figure 6:**
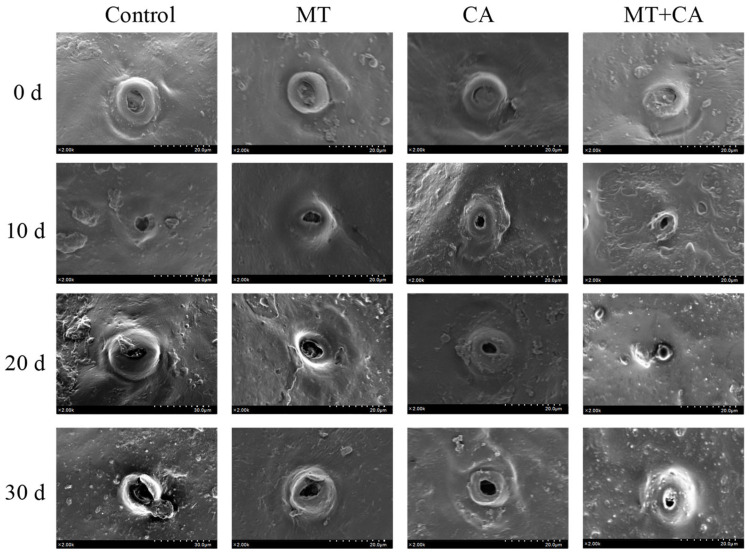
SEM images of MT and/or CA-treated lemon fruits stored at 7–8 °C for 30 d stomata compared to control. The SEM used an analytical mode of secondary electrons with a magnification of 2.00 k, a spot size of 2.00 k × 20.0 μm–30.0 μm, and a working distance of 9.5 mm.

The stomata are the passages through which the water and air permeability of the lemons are invaded, and the opening and closing of the stomata in the guard cells are involved in the regulation of carbon assimilation, respiration, and transpiration [7]. SEM images of lemon microstructure showed no difference in stomata between the different treatment groups on day 0 (Figure 6). With the extension of storage time, the stomatal opening of the Control group became larger and larger, while the stomatal opening of the MT+CA treatment group was significantly smaller than that of the Control group. The results showed that MT+CA could reduce the size of subcutaneous thin-walled cells and delay stomatal expansion, thus inhibiting the reduction in water loss and delaying fruit senescence.

### 3.7. Correlation Analysis

**Figure 7:**
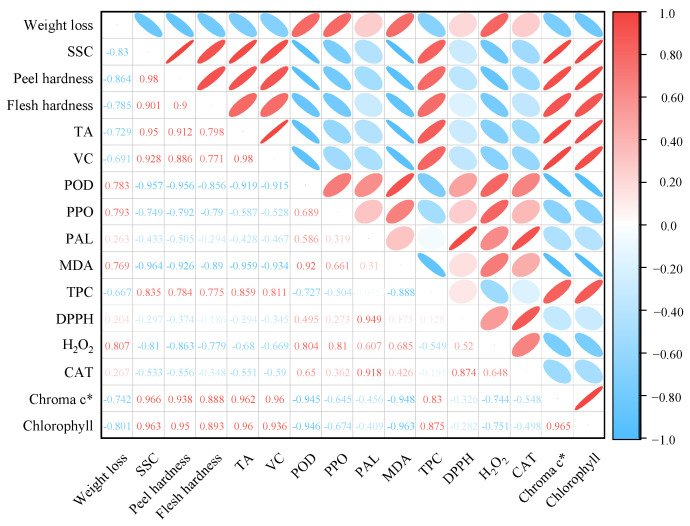
Pearson correlation coefficients for lemon quality traits, including Chroma c*, Chlorophyll, weight loss, SSC, peel hardness, flesh hardness, TA, VC, POD, PPO, PAL, MDA, TPC, DPPH scavenging capacity, H_2_O_2_, CAT, and the following volatile substances: Aromatic (benzene), Broad alcohol, Arom-aliph, Broad oxynitride. Red and blue dots are positive and negative correlations, respectively, and the number shown is the correlation coefficient.

Correlations between textural, antioxidant, membrane peroxidation, and volatiles were analyzed using Pearson analysis in the study by Jiang et al. [33]. Therefore, in this study, Pearson correlation analysis was used to find potential connections between the physical quality characteristics, enzyme activities, and antioxidant activities of lemons during postharvest storage (Figure 7). Chlorophyll content was positively correlated with SSC, peel hardness, flesh hardness, TA, VC, and TPC (r^2^ ≥ 0.88) and negatively correlated with weight loss (r^2^ = −0.80). TPC was positively correlated with chroma c*, chlorophyll, SSC, peel hardness, flesh hardness, TA, and VC (0.78 < r^2^ < 0.88), and negatively correlated with POD (r^2^ = −0.73), PPO (r^2^ = −0.50), and MDA (r^2^ = −0.89) were negatively correlated. POD and PPO were both associated with PAL, MDA, DPPH, and H_2_O_2_, and CAT were positively correlated.

## 4. Conclusions

In conclusion, the postharvest application of MT+CA treatment maintained the firmness of lemon fruits, kept good color, decreased fruit weight loss and TA, VC, and aroma substance loss, and effectively controlled the senescence process of lemon fruits during cold storage. In addition, the combined treatment inhibited the activities of POD, PPO, and CAT and increased PAL activity, the content of TPC, and DPPH scavenging capacity. The levels of H_2_O_2_ and malondialdehyde were reduced, thereby inhibiting the increase in membrane permeability. These effects prolonged the shelf life of lemon fruit, suggesting that MT+CA treatment could be a potential method for improving the quality of lemons under long-term chilling conditions.

## Figures and Tables

**Table 1:** Chroma C* and chlorophyll content of lemons treated with MT and/or CA during 30 days of refrigeration at 7–8 °C.

Time	Treatment	Chroma C*	Chlorophyll (mg/g)
0 d	Control	29.6 ± 0.17 ^a^	44.15 ± 0.72 ^ab^
	MT	30.9 ± 0.18 ^b^	46.33 ± 0.55 ^ab^
	CA	27.8 ± 0.17 ^b^	45.15 ± 1.73 ^b^
	MT+CA	25.3 ± 0.07 ^c^	46.58 ± 1.23 ^a^
10 d	Control	35.2 ± 0.13 ^a^	38.35 ± 2.01 ^b^
	MT	33.1 ± 0.22 ^a^	40.62 ± 1.39 ^ab^
	CA	31.0 ± 0.06 ^ab^	40.19 ± 1.35 ^ab^
	MT+CA	27.9 ± 0.09 ^b^	42.36 ± 1.25 ^a^
20 d	Control	38.4 ± 0.12 ^a^	26.87 ± 1.61 ^c^
	MT	40.7 ± 0.06 ^ab^	29.64 ± 1.58 ^b^
	CA	34.5 ± 0.09 ^b^	28.48 ± 1.59 ^bc^
	MT+CA	34.0 ± 0.08 ^b^	32.22 ± 0.82 ^a^
30 d	Control	49.4 ± 0.11 ^a^	10.37 ± 0.96 ^d^
	MT	47.3 ± 0.15 ^a^	19.62 ± 0.93 ^b^
	CA	36.7 ± 0.27 ^a^	14.11 ± 1.17 ^c^
	MT+CA	34.6 ± 0.06 ^a^	28.47 ± 1.13 ^a^

Note: Data are expressed as SD ± mean. Different letters signify statistically significant (*p* < 0.05) differences between treatments.

## Data Availability

The original contributions presented in the study are included in the article, further inquiries can be directed to the corresponding author.
